# The differentiation of ROR-γt expressing iNKT17 cells is orchestrated by Runx1

**DOI:** 10.1038/s41598-017-07365-8

**Published:** 2017-08-01

**Authors:** Puspa Thapa, Bryce Manso, Ji Young Chung, Sinibaldo Romera Arocha, Hai-Hui Xue, Derek B. Sant’ Angelo, Virginia Smith Shapiro

**Affiliations:** 1 Department of Immunology, Mayo Clinic, College of Medicine, 200 1st Street SW, Rochester, MN 55905 USA; 2 Department of Microbiology and Immunology, University of Iowa, 51 Newton Rd Iowa City, IA, 52242 USA; 3 Department of Pediatrics, Rutgers Robert Wood Johnson Medical School and The Children’s Health Institute of New Jersey, 89 French Street, New Brunswick, NJ 08901 USA

## Abstract

iNKT cells are a unique lineage of T cells that recognize glycolipid presented by CD1d. In the thymus, they differentiate into iNKT1, iNKT2 and iNKT17 effector subsets, characterized by preferential expression of Tbet, Gata3 and ROR-γt and production of IFN-γ, IL-4 and IL-17, respectively. We demonstrate that the transcriptional regulator Runx1 is essential for the generation of ROR-γt expressing iNKT17 cells. PLZF-cre Runx1 cKO mice lack iNKT17 cells in the thymus, spleen and liver. Runx1-deficient iNKT cells have altered expression of several genes important for iNKT17 differentiation, including decreased expression of IL-7Rα, BATF and c-Maf and increased expression of Bcl11b and Lef1. However, reduction of Lef1 expression or introduction of an IL-7Rα transgene is not sufficient to correct the defect in iNKT17 differentiation, demonstrating that Runx1 is a key regulator of several genes required for iNKT17 differentiation. Loss of Runx1 leads to a severe decrease in iNKT cell numbers in the thymus, spleen and liver. The decrease in cell number is due to a combined decrease in proliferation at Stage 1 during thymic development and increased apoptosis. Thus, we describe a novel role of Runx1 in iNKT cell development and differentiation, particularly in orchestrating iNKT17 differentiation.

## Introduction

Invariant natural Killer T (iNKT) cells are innate lymphocytes that express a semi-invariant TCR with an invariant TCRα-chain, Vα14-Jα18, paired with limited TCRβ-chains, Vβ7, Vβ8, or Vβ2. iNKT cells recognizes glycolipids presented on an MHC-like molecule CD1d^1–4^. They share a common developmental precursor with conventional T cells at the double positive (DP) thymocyte stage^5, 6^. Upon positive selection into the iNKT cell lineage at DP stage, iNKT cells go through four sequential developmental stages (Stage 0–3), where Stage 0 is the earliest stage, characterized by high CD24 expression^7^. Unlike conventional T cells that do not proliferate after selection into CD4 or CD8 single positive T cell lineages, iNKT cells undergo a post-selection expansion at Stage 1 where they down-regulate CD24 expression. The intra-thymic proliferation of iNKT cell is highly regulated by molecular networks that involve the transcription factor c-Myc and the other metabolic pathways^8–10^. After proliferating, iNKT cells express an effector/memory phenotype and upregulate the expression of CD44 at Stage 2. Expression of NK receptors such as NK1.1 is turned on at Stage 3, where IL-15 is required for their homeostasis and survival by regulating the expression of Bcl-xL in Stage 3 iNKT cells^11–13^.

Although the traditional linear developmental pathway is used often to examine iNKT cells, iNKT cells differentiate into effector subsets in the thymus within Stages 1 through 3^14, 15^. Their ‘master’ transcription factor PLZF is important for iNKT cell development and effector functions^16, 17^. In the thymus, three subsets that develop are iNKT1, iNKT2 and iNKT17, although there is evidence of other functional subsets in peripheral tissues^14, 18, 19^. iNKT subsets are distinguished by the signature transcription factors they express and the predominant production of cytokines they produce. iNKT1 cells are Tbet^+^ PLZF^lo^, produce IFNγ and are found within NK1.1^+^ Stage 3. iNKT2 cells are PLZF^hi^ Gata3^hi^, produce IL-4 and are found in both Stage 1 and Stage 2. iNKT17 cells are ROR-γt^+^ PLZF^med^, produce IL-17 and are found exclusively in Stage 2^14, 20^.

Various transcriptional regulators and signaling programs have been identified to play a role in regulating iNKT subset differentiation. The mammalian target of rapamycin (mTOR) signaling pathway is crucial for iNKT cell development and differentiation^21^. mTORC1 is essential for differentiation of Tbet expressing iNKT1 while mTORC2 is important for iNKT2 and iNKT17 differentiation^10, 22, 23^. iNKT cells also require autophagy for their survival and the differentiation of iNKT1 cells^24, 25^. The transcriptional repressor NKAP is also required for iNKT cell proliferation and differentiation of ROR-γt expressing iNKT17 cells^26^. The transcription factor Bcl11b is important for restraining the NKT17 differentiation program to allow for differentiation of iNKT2 and iNKT1 cells^27^. The transcription factor Lef1 is also important for iNKT cell proliferation and is crucial for differentiation of iNKT2 cells^28, 29^. Loss of Lef1 leads to an increased proportion and function of iNKT17 cells suggesting Lef1 may restrain iNKT17 differentiation to promote iNKT2 differentiation. The transcription factor BATF is also required for the development of IL-17 producing iNKT cells^30, 31^. Although there is increasing evidence of molecular mechanisms governing iNKT development and differentiation, the interplay of transcription regulators that build molecular networks critical for specific iNKT cell differentiation is not fully understood. Here we show the role of Runx1 in regulating the transcriptional network that drives iNKT17 differentiation.

The Runt related transcription factor Runx1 belongs to the Runx family of transcription factors, and is also known as CBF-α, AML1 or PEBP2alphaB^32^. Runx proteins associate with core binding factor-beta (CBF-β) to bind DNA. The DNA binding domain, *Runt*, binds to a specific core sequence TGTGGNNN (where NNN can either be TTT or TCA)^33^. Runx1 can mediate either gene activation or repression as determined by other transcriptional coactivators or corepressors it binds to, such as HDACs^34^. Runx1 has essential roles in the development and differentiation of several hematopoietic lineages, including being a central player for hematopoiesis, conventional T cell development, and Th17 differentiation^35–39^. Although Runx1 is required for the positive selection of iNKT cells (as demonstrated by Egawa and colleagues’ CD4-cre Runx1 cKO model)^5^, its role in later iNKT cell development and differentiation beyond positive selection is unknown. In this study, we show that Runx1 is absolutely required for iNKT17 differentiation. In addition, we also demonstrate that Runx1 is required for intra-thymic proliferation and survival of iNKT cells.

## Results

### Runx1 is required for iNKT cell development

**Figure 1:**
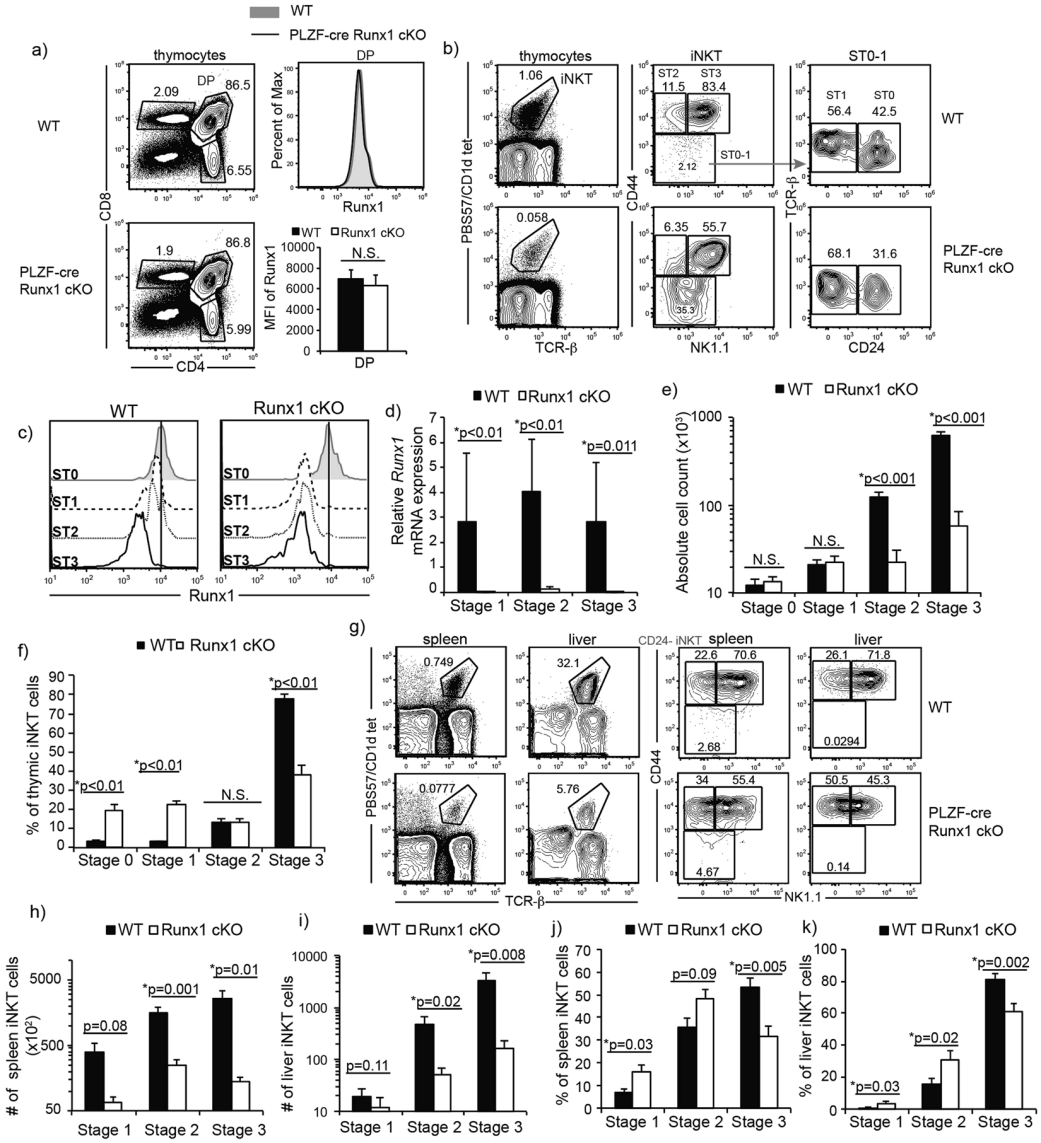
Runx1 is required for iNKT cell development. (**a**) Thymic CD4/CD8 profile in WT and PLZF-cre Runx1 cKO mice. Expression and quantification of Runx1 in DP thymocytes from WT (grey filled) and PLZF-cre Runx1 cKO (black line) mice. Data is representative and calculated from 11 mice per group. (**b**) Examination of thymic iNKT cells in their developmental stages in WT (top) and PLZF-cre Runx1 cKO mice (bottom). iNKT cells were identified using expression of TCR-β and CD1d/PBS57 loaded tetramers. Gated iNKT cells were further characterized into stages using NK1.1, CD44 and CD24 markers: Stage 0 (CD24^+^ CD44^−^ NK1.1^−^) Stage 1 (CD24^−^ CD44^+^ NK1.1^−^) Stage 2 (CD44^hi^ NK1.1^−^) and Stage 3 (CD44^+^ NK1.1^+^). Data is representative of at least 30 mice per genotype from at least 20 independent experiments. (**c**) Expression of Runx1 in Stage 0–3 iNKT cells from WT (grey filled) and PLZF-cre Runx1 cKO (black line) mice. Data is representative of at least 7 mice/genotype from 5 independent experiments. (**d**) Relative mRNA expression of *Runx1* in sorted Stage 1–3 iNKT cells of WT (black bars) and PLZF-cre Runx1 cKO (white bars) mice. Data is normalized to expression of *Runx1* in WT Stage 3 iNKT cells = 1. Data is calculated from 3 WT and 3 PLZF-cre Runx1 cKO mice from 3 independent sorts. (**e,f**) Absolute cell count and percent of thymic iNKT cells at Stage 1, 2 and 3 in WT (black bars) and PLZF-cre Runx1 cKO mice (white bars). Data is calculated from at least 30 mice per group in the age range of 5–12 weeks old. (**g**) Representative frequencies of iNKT cells in spleen and liver of WT and PLZF-cre Runx1 cKO mice. Data is representative of at least 12 mice/genotype from 8 independent experiments in spleen and 9 mice/genotype from 7 independent experiments in liver. (**h-i**) Total Stage 1–3 iNKT cell count from spleen and liver. (**j-k**) Frequency of Stage 1–3 iNKT cells in the spleen and liver. (**h-k**) Data is calculated from at least 12 mice per group from 8 independent experiments in spleen and 9 mice/genotype from 7 independent experiments in liver. All statistical analysis was done using Student’s *t-test*. Means ± S.E.M.

Loss of Runx1 in T cells using the CD4-cre Runx1 cKO mice leads to a complete block in the generation of iNKT cells while development of conventional T cell was normal^5^. Thus, Runx1 is required for the positive selection of DP thymocytes into the iNKT cell lineage. To investigate Runx1’s role in later iNKT cell development and differentiation after positive selection, we generated PLZF-cre Runx1 cKO mice. Expression of PLZF starts at Stage 0 and is required for iNKT cell development. Using a cre-dependent lineage marker, several groups have reported that PLZF-cre is transiently expressed in a precursor to hematopoietic stem cell (HSCs) during embryogenesis, marking ~30–50% of the HSC pool^18, 40^. However, as Runx1 is required for the generation of HSCs, only Runx1-sufficient HSC will develop in PLZF-cre Runx1 cKO mice^41, 42^. This model allows us to study the role of Runx1 specifically in iNKT cells, as conventional T cells do not express PLZF. We confirmed that there was no change in Runx1 expression in DP thymocytes and that conventional T cell development is unaltered in PLZF-cre Runx1 cKO mice (Fig. 1a).

In PLZF-cre Runx1 cKO mice, there is a dramatic alteration in iNKT cell development. The few iNKT cells present were skewed towards earlier stages (Stage 0–1) in development (Fig. 1b). Thus, Runx1 has additional critical roles in iNKT cell development beyond the DP to Stage 0 transition^5^. In PLZF-cre Runx1 cKO mice, Runx1 protein expression does not decrease until Stage 1 in iNKT cells (Fig. 1c). Thus, further analysis will focus on Stage 1 and subsequent stages of iNKT cell development. Deletion of *Runx1* was also examined by q-PCR, where one primer was located within a floxed exon. *Runx1* was efficiently deleted in Stage 1, Stage 2 and Stage 3 iNKT cells from PLZF-cre Runx1 cKO mice (Fig. 1d). Interestingly, WT Stage 3 iNKT cells downregulate Runx1 protein expression although mRNA levels were sustained at levels similar to earlier iNKT cell stages. Examination of the absolute cell count of iNKT cells in thymus of PLZF-cre Runx1 cKO mice revealed a significant reduction (p < 0.001) in cell number of Stage 2 (8.5 fold) and Stage 3 (19.5 fold) iNKT cells (Fig. 1e). The frequency of iNKT cells in Stage 0, Stage 1, Stage 2, and Stage 3 from WT and PLZF-cre Runx1 cKO mice was also quantified (Fig. 1f). There was a 6-fold and 7-fold increase in Stage 0 and Stage 1 iNKT cell frequency respectively, while there was a 2-fold decrease in Stage 3 iNKT cell frequency and no significant change in proportion of Stage 2 iNKT cells. In the periphery, there was also a dramatic decrease of 8-fold and 15-fold in iNKT cell numbers in the spleen and liver, respectively (Fig. 1g). The total cell count and frequency of Stage 1–3 iNKT cells in the spleen and liver was also quantified (Fig. 1h–k). In the spleen, there was a significant decrease in Stage 2 (6.5 fold) and Stage 3 (19 fold) iNKT cell numbers (Fig. 1h). In the liver, there was approximately 9.7-fold and 20-fold decrease in Stage 2 and Stage 3 iNKT cells, respectively (Fig. 1j). This shows that the block in thymic iNKT cell development also reflected in the numbers and proportions of splenic and liver iNKT cells. Hence, Runx1 is important for iNKT cell development after positive selection.

### Runx1 regulates iNKT cell proliferation

**Figure 2:**
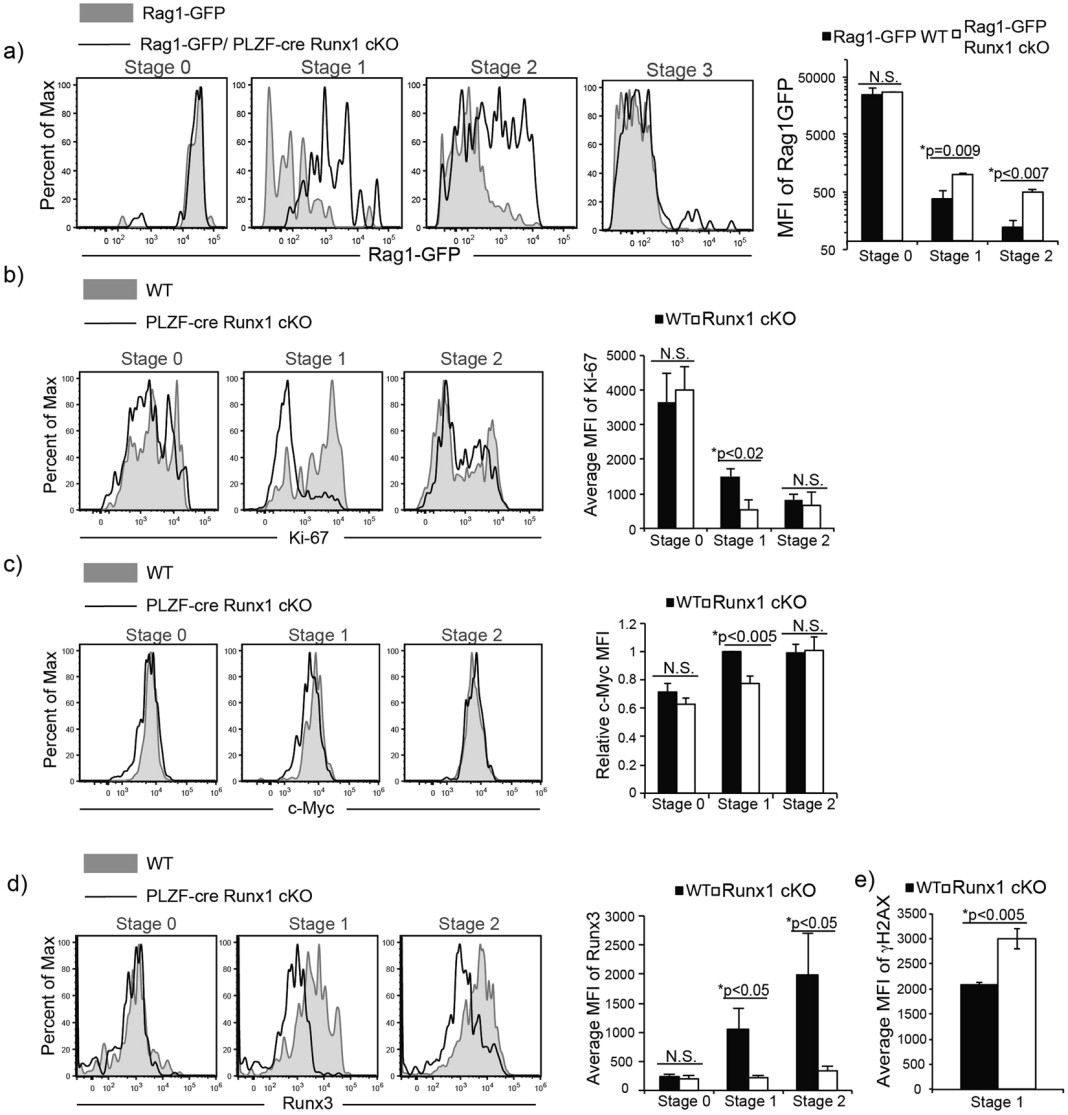
Loss of Runx1 leads to a defect in proliferation and increased DNA damage. (**a**) Expression of GFP in Stage 0–3 iNKT cells of Rag1-GFP (WT) (grey filled) and Rag1-GFP/PLZF-cre Runx1 cKO mice (black line). iNKT cells were gated and staged as described in Fig. 1. Data is representative of at least 3 mice/genotype from 3 independent experiments. Quantification of average mean fluorescent intensity (MFI) of GFP in Stage 0, 1 and 2 iNKT cells from Rag1-GFP (WT) (black bars) and Rag1-GFP/PLZF-cre Runx1 cKO mice (white bars). Data is calculated from at least 3 mice/genotype. (**b**) Intracellular expression of Ki-67 in Stage 0, Stage 1 and Stage 2 iNKT cells of WT (grey filled) and PLZF-cre Runx1 cKO mice (black line). Data is representative of at least 3 mice/genotype from 3 independent experiments. Quantification MFI of Ki-67 in Stage 0–2 in WT (black bar) and PLZF-cre Runx1 cKO (white bar). Data is calculated from 5 WT and 3 PLZF-cre Runx1 cKO mice from 3 independent experiments. (**c**) Intracellular expression of c-Myc in Stage 0–2 iNKT cells of WT (grey filled) and PLZF-cre Runx1 cKO mice (black line). Data is representative of at least 4 mice/genotype from 4 independent experiments. Relative MFI of c-Myc in Stage 0–2 iNKT cells of WT (black bar) and PLZF-cre Runx1 cKO mice (Runx1 cKO) (white bar). Data is normalized to MFI of c-Myc in WT Stage 1 iNKT cells = 1. Data is calculated from 4 mice/genotype from 4 independent experiments. (**d**) Expression of Runx3 in Stage 0–2 of WT (grey filled) and PLZF-cre Runx1 cKO (black line). Data is representative of at least 3 mice/genotype from 3 independent experiments. Quantification of MFI of Runx3 in Stage 0–2 in WT (black bar) and PLZF-cre Runx1 cKO (white bar). Data is calculated from 3 WT and 3 PLZF-cre Runx1 cKO mice from 3 independent experiments. (**e**) Average MFI of γH2AX was calculated for Stage 1 iNKT cells in WT (black bars) and PLZF-cre Runx1 cKO mice (white bars). Data is calculated from 3 mice/genotype from 3 independent experiments. All statistical analysis was done using Student’s *t-test*. Means ± S.E.M.

After positive selection into lineage, unlike conventional T cells, iNKT cells undergo a burst of proliferation. To determine if Runx1-deficient iNKT cells have a defect in proliferation leading to a significant reduction in absolute cell number, we generated Rag1-GFP/PLZF-cre Runx1 cKO mice. The use of Rag1-GFP reporter mice to examine the rate of proliferation in iNKT cell has been described previously^26^. Briefly, GFP is knocked into one allele of the *Rag1* locus^43^. Transcription of *Rag1* stops at the DP thymocyte stage after successful rearrangement of TCR-α chain. However, GFP protein is stable with a long half-life^44^. In the periphery, Rag1-GFP persists in conventional T cells (which do not proliferate after DP thymocyte stage) for up to 3 weeks, and is used to mark recent thymic emigrants (RTE)^44^. However, iNKT cells undergo a burst of proliferation at Stage 1, leading to the dilution of GFP, allowing us to directly measure proliferation *in vivo*. Prior to the proliferative burst, the expression of Rag1-GFP reporter in Stage 0 iNKT cells of Rag1-GFP/PLZF-cre Runx1 cKO mice was equivalent to Stage 0 iNKT cells of Rag1-GFP (WT) mice. However, at Stage 1 and Stage 2, the Rag1-GFP reporter expression was significantly higher in iNKT cells of Rag1-GFP/PLZF-cre Runx1 cKO mice compared to Rag1-GFP (WT) mice (Fig. 2a), indicating a reduced rate of proliferation in the absence of Runx1. The expression of Rag1-GFP was 2.7 fold and 4 fold increased in Stage 1 and Stage 2 iNKT cells of Rag1-GFP/PLZF-cre Runx1 cKO, respectively, compared to Rag1-GFP WT mice. The expression of Ki-67, a proliferative capacity marker, was also significantly reduced by 3 fold in Runx1-deficient Stage 1 iNKT cells but not Stage 0 and Stage 2 iNKT cells (Fig. 2b). Similarly, the transcription factor c-Myc is required for iNKT cell proliferation and was reduced by 30% (p < 0.005) in Runx1-deficient Stage 1 iNKT cells (Fig. 2c). In HSCs, loss of both Runx1 and Runx3 lead to a defect in DNA repair resulting in increased DNA damage^42^. Thus, if Runx3 was also reduced in Runx1-deficient iNKT cells, there may be increased DNA damage which could contribute to the block in proliferation. The expression of Runx3 was significantly reduced by ~5 fold in Runx1-deficient Stage 1 and Stage 2 iNKT cells (Fig. 2d). To determine if loss of Runx1 with reduced expression of Runx3 led to increased DNA damage during proliferation of iNKT cells, we examined the expression of γH2AX by flow cytometry. Runx1-deficient Stage 1 iNKT cells had a statistically significant increase (~1.5 fold) in γH2AX expression compared to WT, demonstrating increased DNA damage (Fig. 2e). Hence, Runx1 is required for iNKT cell proliferation during thymic development.

### Runx1 is required for iNKT cell survival

**Figure 3:**
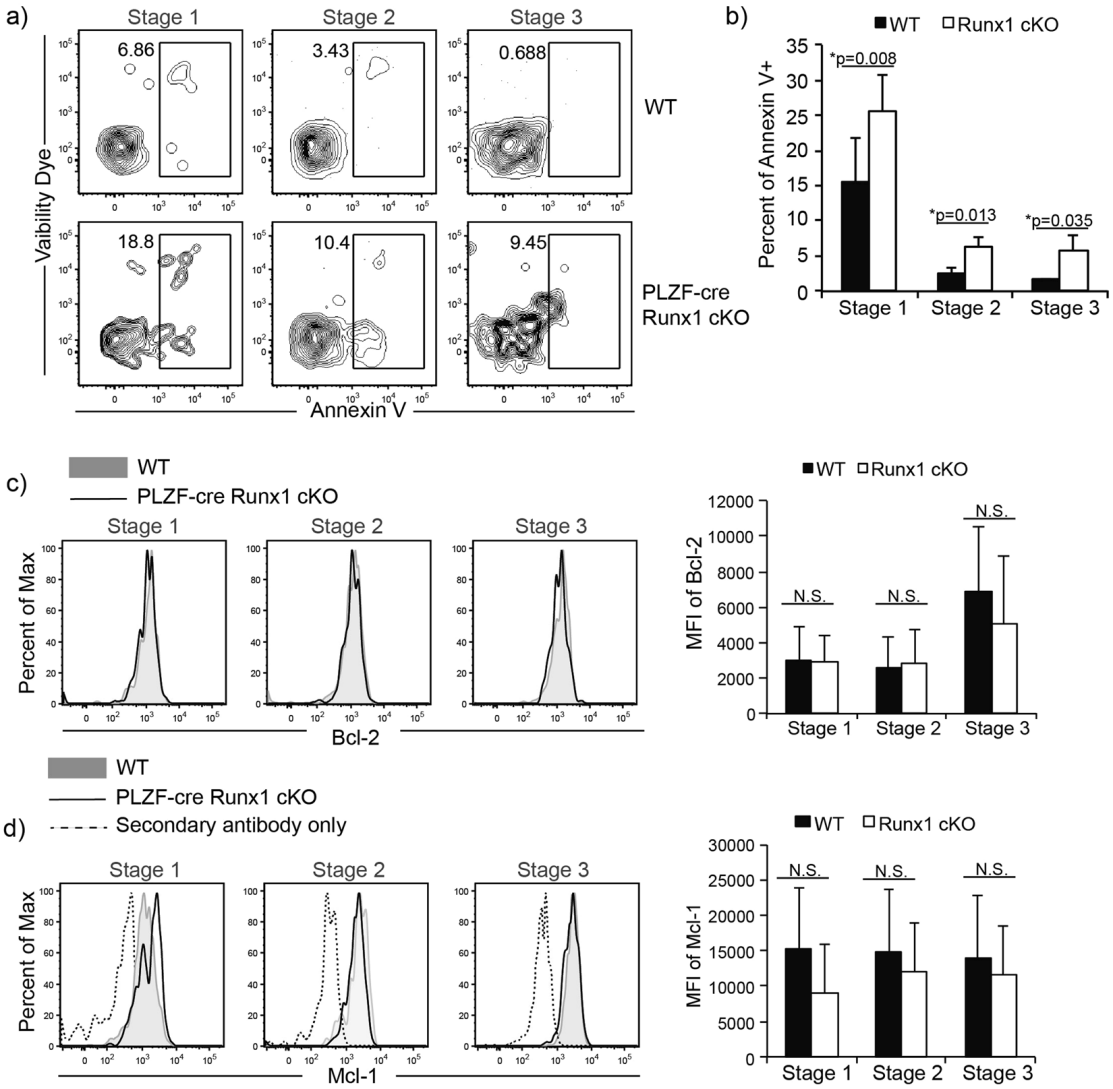
Runx1-deficient iNKT cells exhibit increased apoptosis but normal expression of pro-survival genes Bcl-2 and Mcl-1. (**a**) Representative frequency of apoptotic (Annexin V^+^) Stage 1–3 iNKT cells in WT (top) and PLZF-cre Runx1 cKO mice (bottom). Gating for iNKT cells and their developmental stages were defined as described in Fig. 1. Data is representative of at least 7 mice/genotype from 5 different experiments. (**b**) Quantification of apoptotic Stage 1–3 iNKT cells in WT (black bars) and PLZF-cre Runx1 cKO mice (white bars). Data was calculated from at least 7 mice/genotype from 5 different experiments. (**c**) Intracellular expression of pro-survival gene Bcl-2 in Stage 1–3 iNKT cells from WT (grey filled) and PLZF-cre Hdac3 cKO (black line). Data is representative of at least 3 mice/genotype from 3 different experiments. Quantification of MFI of Bcl-2 in Stage 1–3 iNKT cells in WT (black bars) and PLZF-cre Runx1 cKO mice (white bars). Data was calculated from 3 mice/genotype. (**d**) Intracellular expression of pro-survival gene Mcl-1 in Stage 1-3 iNKT cells from WT (grey filled) and PLZF-cre Hdac3 cKO (black line). Secondary only control for Mcl-1 is shown as dashed line. Data is representative of at least 3 mice/genotype from 3 different experiments. Quantification of MFI of Mcl-1 in Stage 1-3 iNKT cells in WT (black bars) and PLZF-cre Runx1 cKO mice (white bars). Data was calculated from 5 WT and 3 PLZF-cre Runx1 cKO mice. All statistical analysis was done using Student’s *t-test*. Means ± S.E.M.

To address whether increased cell death may also contribute to decreased iNKT numbers in PLZF-cre Runx1 cKO mice, we examined apoptosis by Annexin V and a fixable viability dye (FVD). Stage 1, Stage 2 and Stage 3 Runx1-deficient iNKT cells had an increased proportion of apoptotic or dead cells compared WT iNKT cells (Fig. 3a). The frequency of apoptotic Stage 1–3 iNKT cells in PLZF-cre Runx1 cKO mice was significantly increased by 1.6 fold in Stage1, 2.4 fold in Stage 2 and 3.7 fold in Stage 3 as compared to WT (Fig. 3b). However, no differences were observed in expression of pro-survival molecules Bcl-2 and Mcl-1 in Runx1-deficient iNKT cells as compared to WT iNKT cells (Fig. 3c,d). Thus loss of Runx1 results in increased cell death, which is not due to decreased expression of the Bcl-2 family molecules. Stage 3 iNKT cells require IL-15 signaling for their homeostasis and survival^12, 13^. To determine if loss of Runx1 leads to dysregulated homeostasis, we examined the expression of IL-15 receptor subunits (IL-15Rα, IL-2Rβ, γc) and their target, pro-survival molecule Bcl-xL. The expression of the IL-15 receptor subunits and Bcl-xL was not altered in Stage 3 iNKT cells of PLZF-cre Runx1 cKO mice compared to WT (Supplemental Fig. 1). This suggests that the 9-fold decrease in Stage 3 iNKT cell numbers in PLZF-cre Runx1 cKO mice (Fig. 1e) is not due to a defect in IL-15 mediated homeostasis. Together, decreased proliferation and increased apoptosis in the absence of Runx1 contribute to the severe decrease in iNKT cell numbers in PLZF-cre Runx1 cKO mice.

### Runx1 is required for the differentiation of ROR-γt expressing NKT17 cells

**Figure 4:**
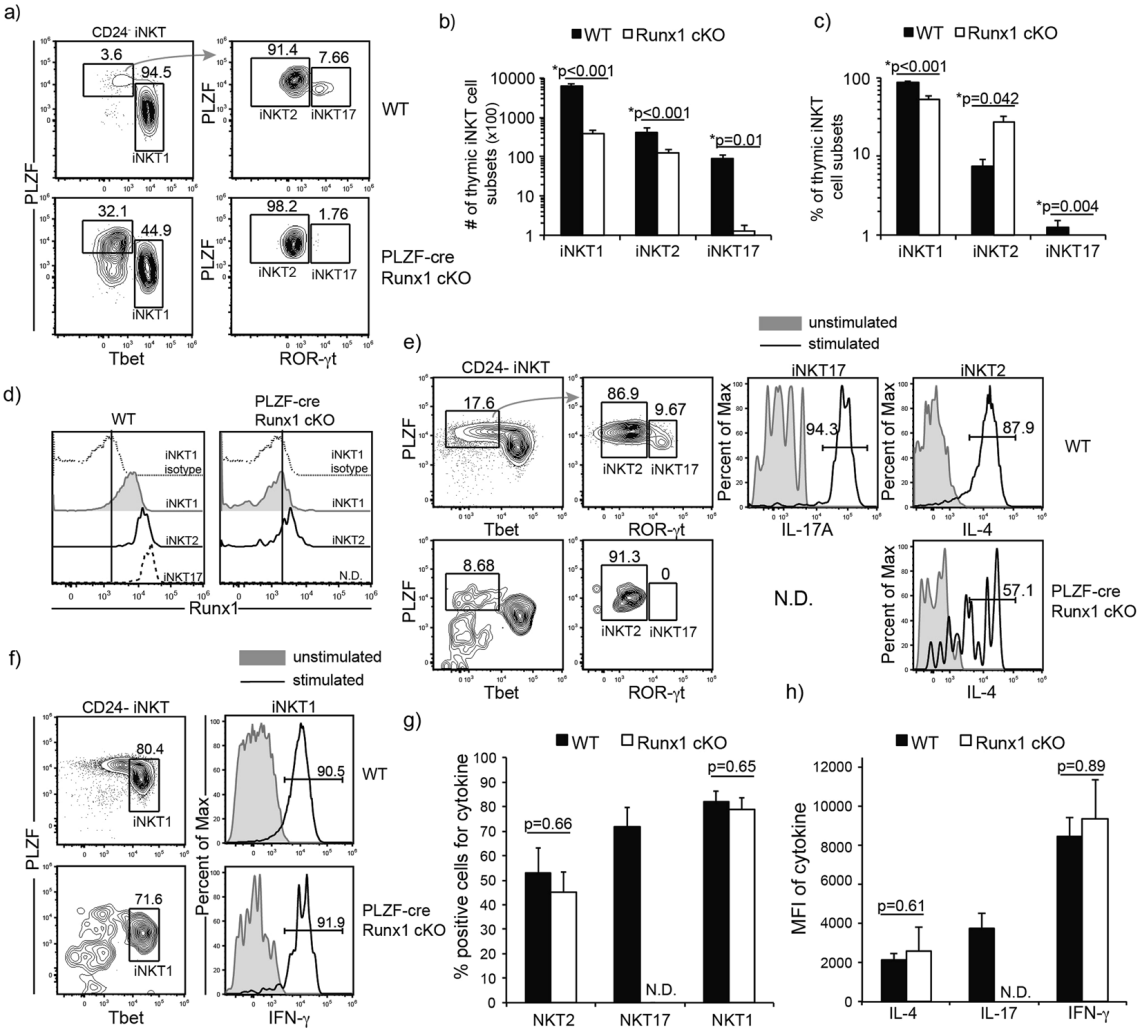
Runx1 is required for differentiation of ROR-γt expressing iNKT17 cells. (**a**) Representative frequency of iNKT subsets, iNKT1, iNKT2 and iNKT17 in WT (top) and PLZF-cre Runx1 cKO (bottom) mice. Functional subsets were defined by expression of PLZF, Tbet, ROR-γt. iNKT1 (Tbet^+^ PLZF^lo^), iNKT2, (PLZF^hi^ ROR-γt^−^ Tbet^−^) and iNKT17 (ROR-γt^+^ PLZF^med^ Tbet^−^). (**b**) Absolute cell number of iNKT1, iNKT2 and iNKT17 cells in WT (black bars) and PLZF-cre Runx1 cKO (white bars) mice. (**c**) Percent of thymic iNKT1, iNKT2 and iNKT17 cells in WT (black bars) and PLZF-cre Runx1 cKO (white bars) mice. (**a-c**) Data is representative and calculated from 22 WT and 12 PLZF-cre Runx1 cKO mice. (**d**) Expression of Runx1 in thymic iNKT1, iNKT2 and iNKT17 in WT and PLZF-cre Runx1 cKO mice with isotype control for WT iNKT1. Data is representative of 7mice/genotype. (**e**) Production of IL-4 and IL-17A by iNKT2 and iNKT17 cells in WT and IL-4 in PLZF-cre Runx1 cKO mice. Production of IL-17A by Runx1-deficient iNKT cells is not determined (N.D.) due to absence of ROR-γt expressing iNKT17 cells. Thymocytes were stimulated for 6hr with PMA/ionomycin, and then examined for cytokine productions using flow cytometry. (**f**) Production of IFN-γ by iNKT1 cells in WT (top) and PLZF-cre Runx1 cKO (bottom) mice. Thymocytes were stimulated for 6hr with PMA/ionomycin, and then examined for cytokine production using flow cytometry. (**g**) Frequency of cells positive for cytokine production (IL-4, IL-17A and IFN-γ) in stimulated iNKT2, iNKT17, and iNKT1 respectively in WT (black bars) and PLZF-cre Runx1 cKO (white bars) mice. Production of IL-17A by Runx1-deficient iNKT cells is not determined (N.D.) due to absence of ROR-γt expressing iNKT17 cells. (**h**) Quantification of MFI of cytokine (IL-4, IL-17A and IFN-γ) produced by stimulated iNKT2, iNKT17, and iNKT1, respectively in WT (black bars) and PLZF-cre Runx1 cKO (white bars) mice. Production of IL-17A by Runx1-deficient iNKT cells is not determined (N.D.) due to absence of ROR-γt expressing iNKT17 cells. (**e-h**) Data is representative and calculated from 8 WT and 4 PLZF-cre Runx1 cKO mice. All statistical analysis was done using Student’s *t-test*. Means ± S.E.M.

Although thymic iNKT cells were traditionally characterized using a linear developmental pathway, it is now established that iNKT cells also differentiate into effector subsets after Stage 0 in the thymus. Thymic iNKT cells differentiate into iNKT1, iNKT2 and iNKT17, although differentiation into other subsets such as iNKT_FH_ and iNKT10 have been described in the periphery^18, 19, 45^. iNKT1, iNKT2 and iNKT17 cells are characterized by the preferential expression of transcription factors. iNKT1 cells are Tbet^+^ PLZF^lo^ ROR-γt^−^, iNKT2 cells are PLZF^hi^ ROR-γt^−^ Tbet^-^ and iNKT17 cells are ROR-γt^+^ PLZF^med^ Tbet^−^. iNKT1, iNKT2 and iNKT17 preferentially produce IFN-γ, IL-4 and IL-17A, respectively^14, 46^. In PLZF-cre Runx1 cKO mice, the proportion of iNKT1 (Tbet^+^ PLZF^lo^) cells was reduced (1.6 fold), while the proportion of Tbet^−^ PLZF^+^ (containing iNKT2 and iNKT17) cells was increased by 3 fold. Examination of ROR-γt expression in the PLZF^+^ Tbet^−^ gate to delineate iNKT17 from iNKT2 revealed the absence of ROR-γt expressing iNKT17 cells and increased proportion of PLZF^hi^ ROR-γt^−^ iNKT2 cells (Fig. 4a). Although the frequency of iNKT2 cells was increased, while the frequency of iNKT1 and iNKT17 cells was decreased in the PLZF-cre Runx1 cKO mice, the absolute count of thymic iNKT cell subsets revealed a statistically significant decrease in numbers in all three subsets. In the thymus, iNKT1 were decreased 16 fold, iNKT2 were decreased 3 fold and iNKT17 were decreased 60 fold in the PLZF-cre Runx1 cKO mice as compared to WT (Fig. 4b,c). The alteration in the frequency of thymic iNKT cell subsets (increased iNKT2 cells and decreased iNKT1 cells, absence of iNKT17 cells) in the PLZF-cre Runx1 cKO mice was also mirrored in the spleen and liver. Moreover, similar to thymic iNKT cell counts, there was a severe reduction in the number of iNKT1 (decreased 25 fold in the spleen and 12 fold in the liver) and iNKT2 cells (decreased 8 fold in the spleen and 29 fold in the liver), while there was a complete absence of iNKT17 cells in the periphery, spleen and liver (Supplemental Fig. 2). Thus, the severe defects in iNKT1, iNKT2 and iNKT17 cell generation in the thymus lead to similar reductions in the periphery. The expression of Runx1 also varied by effector subset. In WT mice, expression was highest in iNKT17 cells, while iNKT2 cells had intermediate Runx1 expression, and iNKT1 cells expressed the least Runx1 (Fig. 4d). Similar to our earlier analysis of Runx1 protein expression in PLZF-cre cKO mice, there was a large reduction in Runx1 protein expression in iNKT1 and iNKT2 cells (while iNKT17 cells, which are not generated in these mice, could not be examined) (Fig. 4d). Loss of Runx1 did not alter or significantly change the cytokine production of IFN-γ and IL-4 by iNKT1 and iNKT2, respectively (Fig. 4e,f). Quantification of either the percent positive cells for IFN-γ and IL-4 cytokine production and quantification of MFI of IFN-γ and IL-4 by stimulated iNKT1 and iNKT2 cells, confirmed that there was not a statistically significant change in cytokine production by Runx1-deficient iNKT1 and iNKT2 cells (Fig. 4g,h). Thus, Runx1 is essential for the differentiation of ROR-γt expressing iNKT17 cells. While there are fewer iNKT1 and iNKT2 cells present in PLZF-cre Runx1 cKO mice, Runx1 deficiency did not alter cytokine production by iNKT1 or iNKT2 cells.

### Runx1 regulates the transcription program that governs iNKT17 differentiation

**Figure 5:**
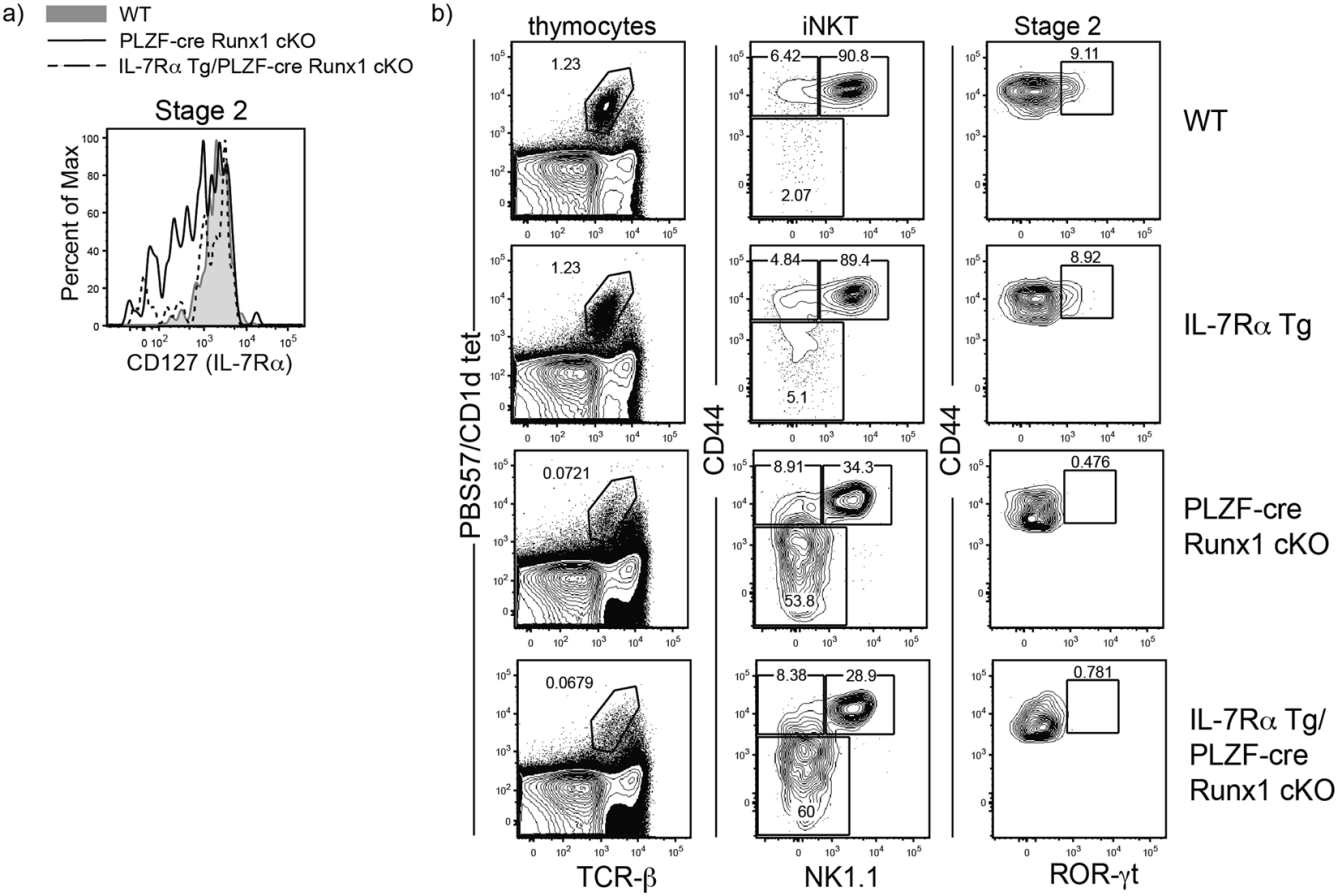
Transgenic expression of IL-7Rα in PLZF-cre Runx1 cKO mice does not rescue block in iNKT17 differentiation. (**a**) Expression of IL-7Rα in Stage 2 iNKT cells from WT (grey tint) and PLZF-cre Runx1 cKO (black line) and IL-7Rα Tg/PLZF-cre Runx1 cKO (dashed line) mice. Data is representative of 3 mice/genotype from 2 independent experiments. (**b**) FACS analysis of iNKT cell development in WT, IL-7Rα Tg, and IL-7Rα Tg/PLZF-cre Runx1 cKO mice. Analysis of iNKT cells was performed as described in Fig. 1. Data is representative of 3 mice/genotype from 2 independent experiments.

**Figure 6:**
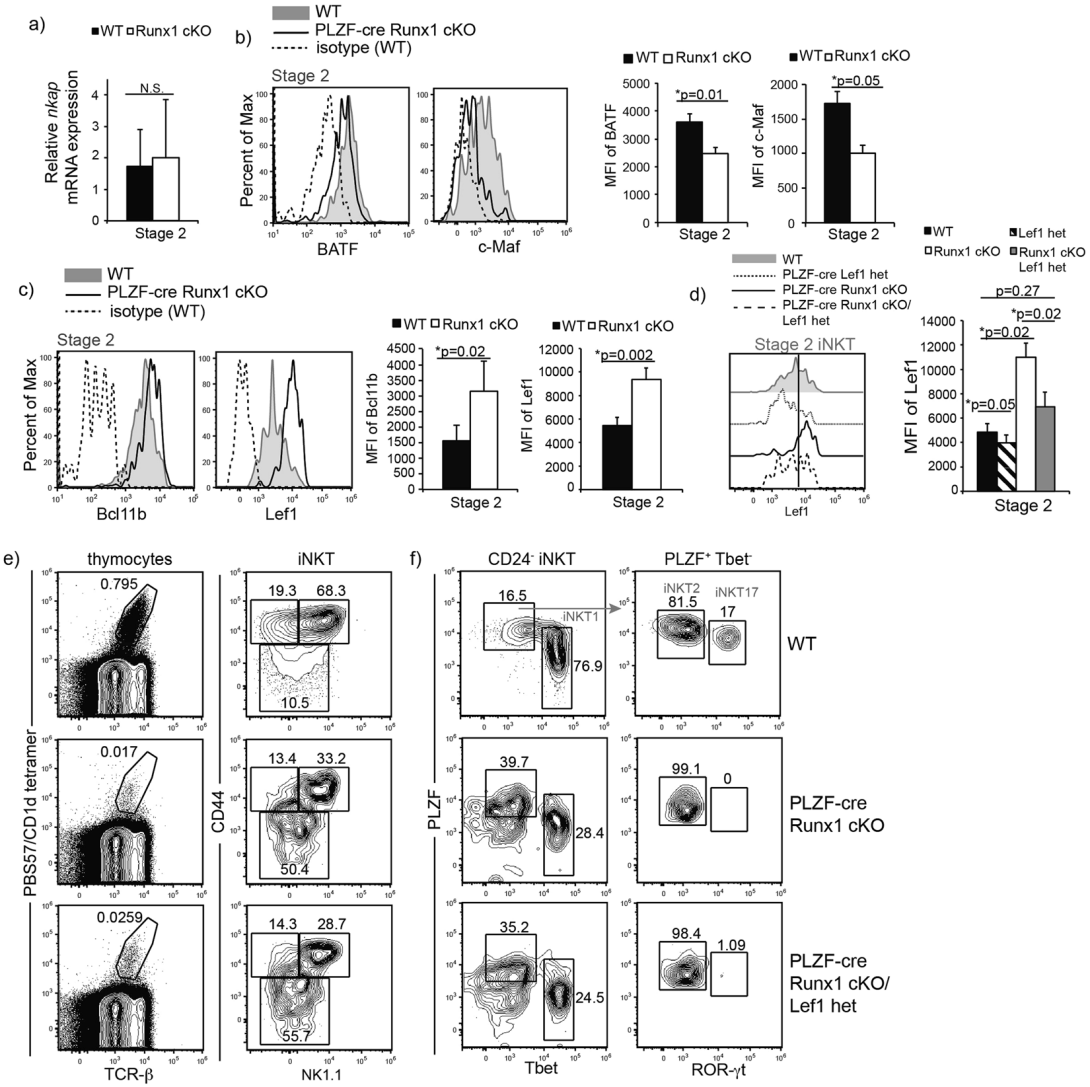
Runx1-deficient iNKT cells exhibit a dysregulated transcriptional program required for iNKT17 cell differentiation. **(a**) Relative mRNA expression of *nkap* in sorted Stage 2 iNKT cells of WT (black bar) and PLZF-cre Runx1 cKO mice (white bar). Data is calculated from 3 WT and 3 PLZF-cre Runx1 cKO mice. (**b**) Intracellular expression of BATF and c-Maf in Stage 2 iNKT cells of WT (grey filled), PLZF-cre Runx1 cKO mice (black line) and isotype control (dashed line). Quantification of MFI for BATF and c-Maf in Stage 2 iNKT cells of WT (black bar) and PLZF-cre Runx1 cKO mice (white bar). Data is representative (histogram overlays) and calculated from at least 11 mice/genotype from 5 independent experiments. (**c**) Intracellular expression of Bcl11b and Lef1 in Stage 2 iNKT cells in WT (grey filled) and PLZF-cre Runx1 cKO mice (black line). Quantification of MFI for Bcl11b and Lef1 in Stage 2 iNKT cells of WT (black bar) and PLZF-cre Runx1 cKO mice (white bar). Data is representative (histogram overlays) and calculated from at least 6 mice/genotype from 4 independent experiments for Bcl11b and 11 mice/genotype from 6 independent experiments for Lef1. (**d**) Lef1 expression in Stage 2 iNKT cells from WT (grey filled), PLZF-cre Lef1 het (dotted line), PLZF-cre Runx1 cKO (black line) and PLZF-cre Runx1 cKO/Lef1 het (dashed line) mice. Quantification of MFI of Lef1 in Stage 2 iNKT cells from WT (black bar), PLZF-cre Runx1 cKO (white bar) and PLZF-cre Runx1 cKO/Lef1 het (grey bar) mice and PLZF-cre Lef1 het (striped bar) mice. Data is representative (histogram overlay) and calculated from 4 mice/genotype from 4 independent experiments. (**e**) Development of iNKT cells in WT, PLZF-cre Runx1 cKO and PLZF-cre Runx1 cKO/Lef1 het mice. Gating was performed as described in Fig. 1. Data is representative of 4 mice/genotype from 4 independent experiments. (**f**) Representative frequency of iNKT subsets: iNKT1, iNKT2 and iNKT17 in WT, PLZF-cre Runx1 cKO and PLZF-cre Runx1 cKO/Lef1 het mice. Subsets gating were performed as described in Fig. 5. Data is representative of 4 mice/genotype from 4 independent experiments. All statistical analysis was done using Student’s *t-test*. Means ± S.E.M.

The linear developmental pathway and differentiation pathway of iNKT cells can be aligned together to understand the dynamics of the development and differentiation occurring synchronously. iNKT1 cells are NK1.1^+^ Stage 3 iNKT cells; iNKT2 cells are found in both Stage 1 and Stage 2 iNKT cells where PLZF expression is the highest, whereas iNKT17 are found exclusively in Stage 2. As the loss of Runx1 leads to a defect in generating ROR-γt expressing iNKT17 cells, we utilized the linear developmental gating scheme (Stage 2) to examine Runx1 regulation of other genes important for iNKT17 differentiation. Although IL-15 is important for iNKT1/Stage 3 cell homeostasis, IL-7 is exclusively required for the differentiation of ROR-γt expressing iNKT17 cells^47^. The expression of IL-7Rα was reduced in Stage 2 Runx1-deficient iNKT cells (Fig. 5a). To determine whether lower expression of IL-7Rα could be responsible for the defect in iNKT cell generation in PLZF-cre Runx1 cKO mice, an IL-7Rα transgene was crossed into PLZF-cre Runx1 cKO mice to improve the expression of IL-7Rα in Stage 2 Runx1-deficient iNKT cells (Fig. 5a). However, the defect in the generation of iNKT17 cells was not rescued in IL-7Rα Tg/PLZF-cre Runx1 cKO mice (Fig. 5b). Interestingly, IL-7’s regulation on iNKT17 cells was independent of STAT signaling but was dependent on the PI3K/AKT/mTOR pathway^47^. To test this, we examined whether there were differences in expression of mTOR, phosphoAKT (Ser374) and phosphoS6 kinase in Runx1-deficient Stage 2 iNKT cells. However, this analysis did not reveal any alteration in the expression of these molecules in Runx1-deficient Stage 2 iNKT cells as compared to WT Stage 2 iNKT cells (Supplemental Fig. 2). Therefore, the defect in iNKT17 generation in the absence of Runx1 is not due to lower levels of IL-7Rα expression. The transcriptional repressor NKAP is required for the differentiation of ROR-γt expressing iNKT17 cells^26^. However, the expression of *nkap* was not altered in Runx1-deficient Stage 2 iNKT cells compared to WT iNKT cells (Fig. 6a), demonstrating that Runx1-mediated regulation of iNKT17 differentiation is independent of NKAP. The ectopic expression of PLZF in conventional T cells lead to an increase in IL-17A producing T cells^48^, suggesting that PLZF may regulate ROR-γt expressing cells. Previously, we showed that transgenic expression of PLZF in NKAP-deficient iNKT cells rescued the generation and function of ROR-γt expressing iNKT17 cells^26^. To determine if ectopic expression of PLZF in Runx1-deficient iNKT cells could also restore generation of iNKT17 cells, we generated Lck-PLZF Tg/PLZF-cre Runx1 cKO mice. However, the transgenic expression of PLZF also did not rescue the defect in differentiation of iNKT17 cells (Supplemental Fig. 3). To understand the molecular mechanism of how Runx1 regulates iNKT17 differentiation, we examined several transcription regulators known to alter either the differentiation of iNKT17 cells or *rorc* expression in Th17 differentiation. The transcription factor c-Maf is important for *rorc* expression in Th17 differentiation^49^ and is highly expressed in iNKT17 cells^20^. The transcription factor, BATF is also required for the differentiation of iNKT17 cells^31^. Runx1-deficient Stage 2 iNKT cells had decreased expression of both BATF and c-Maf (Fig. 6b) and quantification of the MFI revealed a statistically significant decrease in the expression of BATF (1.5 fold) and c-Maf (2 fold) (Fig. 6b). The transcription factors Bcl11b and Lef1 restrain the differentiation of iNKT17 cells as iNKT cells deficient for either transcription factor had increased frequencies of ROR-γt expressing iNKT17 cells^27, 29^. Interestingly, in PLZF-cre Runx1 cKO mice, there was a statistically significant increased expression of both Bcl11b (2 fold) and Lef1 (2 fold) in Stage 2 iNKT cells (Fig. 6c). Taken together, Runx1 plays an essential role in regulating the several genes previously implicated in iNKT17 differentiation. To understand if Runx1 mediates Lef1 expression to direct proper iNKT17 differentiation, we decreased Lef1 expression in Runx1-deficient iNKT cells by generating PLZF-cre Lef1^fl/wt^ Runx1^fl/fl^ mice (Fig. 6d). However, reduction of Lef1 expression in Runx1-deficient iNKT cells did not rescue the block in iNKT cell development (Fig. 6e) or the defect in iNKT17 differentiation (Fig. 6f). Therefore, Runx1 controls iNKT17 differentiation by regulating several genes involved in the differentiation of iNKT17 cells, and alterations in the expression of a single factor (Lef1 or IL-7Rα) was insufficient to restore iNKT17 generation.

## Discussion

Runx1 is required for the positive selection of DP thymocytes into the iNKT cell lineage, as demonstrated using CD4-cre Runx1 cKO mice^5^. However, it was unknown whether Runx1 plays additional roles in subsequent development and differentiation of iNKT cells. In this study, we show that Runx1 is critical in regulating proliferation and survival of iNKT cells, as well as the differentiation of ROR-γt expressing iNKT17 cells. In PLZF-cre Runx1 cKO mice, there is a dramatic decrease in Stage 2 (8.5 fold) and Stage 3 (19.5 fold) iNKT cell numbers. Loss of Runx1 leads to a defect in proliferation, with reductions in Ki-67 (3 fold) and c-Myc (30%) and an increase in γH2AX (1.5 fold) expression. Furthermore, Runx1-deficient iNKT cells exhibit increased cell death that is not due to altered expression of the pro-survival molecules Bcl-2 and Mcl-1. Loss of Runx1 in iNKT cells leads to a defect in the differentiation and function of ROR-γt expressing iNKT17 cells. Runx1-deficient iNKT cells exhibit changes in the expression of several genes required for the proper differentiation of iNKT17 cells. There is a decreased expression of c-Maf (2 fold) and BATF (1.5 fold), while there is an increased expression of Bcl11b (2 fold) and Lef1 (2 fold). Thus, Runx1 is a key regulator required for differentiation of iNKT17 cells.

Proliferation generates DNA damage which is immediately repaired upon detection at defined cell cycle checkpoints^50^. Runx1 and Runx3 mediate DNA damage repair in hematopoietic stem cells, and loss of both Runx1 and Runx3 lead to increased genetic instability with increased levels of γH2AX^42^. Runx1-deficient iNKT cells have reduced expression of Runx3 (5 fold) and increased γH2AX (1.5 fold) staining. Furthermore, in the PLZF-cre Runx1 cKO/Rag1-GFP mice, there is a defect in the rate of proliferation. This suggests that during the burst of proliferation at Stage 1, loss of Runx1 in iNKT cells coupled with decreased Runx3 expression leads to increased DNA damage. The increase in DNA damage may also contribute to the increased cell death observed in PLZF-cre Runx1 cKO iNKT cells (Fig. 3a,b). However, Runx1-deficient Stage 1 iNKT cells also exhibit reduced expression of c-Myc and the proliferative capacity marker Ki-67, demonstrating that it is not simply increased DNA damage which is responsible for the decreased proliferation. It is unclear whether Runx1 directly regulates c-Myc expression in iNKT cells. It has been reported that Runx1 can directly bind and repress c-Myc promoter in Jurkat T cells and murine primary hematopoietic cells^51^. However, as Runx proteins can function as either activators or repressors depending on its binding partners and cellular contexts, it does not exclude the possibility that Runx1 is a positive regulator of c-Myc in iNKT cells.

The Runx family of transcription factors consists of three members, Runx1, Runx2 and Runx3. In CD4-cre Runx3 cKO mice, there is minimal effect on the development of iNKT cells and iNKT differentiation^52^. Whereas in the CD4-cre Runx1 cKO mice, there is a complete block in iNKT cell development, suggesting that other Runx transcription factors cannot compensate for loss of Runx1^5^. However, examination of any functional redundancy between Runx3 and Runx1 during later iNKT cell development and differentiation will need to await generation of PLZF-cre Runx1/Runx3 dKO mice.

Previously, we demonstrated that the transcriptional repressor NKAP is required for the positive selection of DP thymocytes into the iNKT cell lineage^53^. NKAP is also required for iNKT cell proliferation and the differentiation of iNKT17 cells^26^. Transgenic expression of PLZF (Lck-PLZF) in PLZF-cre NKAP cKO, restored iNKT17 differentiation defect but not proliferation^26^. Runx1 is also required for positive selection of DP thymocytes^5^ and in this study we have demonstrated additional roles for Runx1 in iNKT cell proliferation, survival and iNKT17 differentiation. However, Runx1 and NKAP have different roles in mediating iNKT17 differentiation, as *nkap* mRNA expression is normal and transgenic expression of PLZF in PLZF-cre Runx1 cKO mice did not rescue the defect in iNKT17 differentiation as it did in PLZF-cre NKAP cKO mice. Furthermore, expression of Runx1 is unaltered in the PLZF-cre NKAP cKO mice (P.T. and V.S.S., unpublished data). Therefore, Runx1 and NKAP regulate iNKT17 differentiation independently of each other.

Runx1 can induce *rorc* expression and associate with ROR-γt during conventional Th17 differentiation^39^. However, Runx1’s role in iNKT17 differentiation has not been defined. The transcriptional program governing the differentiation of ROR-γt expressing iNKT17 cells involve the interplay of various transcriptional regulators acting as activators and repressors. IL-7 signaling is crucial exclusively for iNKT17 homeostasis and survival. IL-7-mediated regulation of iNKT17 homeostasis was independent of STAT signaling but was dependent on the PI3K/AKT/mTOR pathway^47^. BATF is important for IL-17A producing CD4 T cells (Th17) and iNKT17 cells^31, 54^. The transcription factor c-Maf also regulates the differentiation of Th17 by associating with Sox5 to directly bind and activate the *rorc* promoter in conventional CD4 T cells^49, 55^. However, the functional role of c-Maf is not understood in iNKT17 differentiation, although a recent study reported high mRNA expression of *maf* in iNKT17 cells by RNA-seq^20^. The transcriptional repressor NKAP is also critical for iNKT cell proliferation and differentiation of iNKT17 cells^26^. While BATF, c-Maf and NKAP positively regulate iNKT17 differentiation; the transcription factors Bcl11b and Lef1 restrain iNKT17 differentiation. Deficiency in either factor in iNKT cells led to an increased frequency of iNKT17 cells^27, 29^. Although, Runx1 can induce *rorc* expression and associate with ROR-γt during Th17 differentiation^39^, its role in iNKT17 differentiation has not been defined. It is unlikely that the only role for Runx1 in iNKT cell differentiation is by directly regulating *rorc* expression, as we have shown that the loss of Runx1 leads to statistically significant dysregulation of the expression of transcription factors (BATF, c-Maf, Bcl11b and Lef1) involved in the iNKT17 differentiation program. Although the expression of NKAP was not altered, Runx1-deficient Stage 2 iNKT cells have decreased expression of the positive regulators, BATF and c-Maf, while the expression of negative regulators, Bcl11b and Lef1, is increased. Furthermore, decreasing Lef1 expression in Runx1-deficient iNKT cells did not correct the iNKT17 differentiation and IL-17A production defect, suggesting that restoration of a part of the dysregulated transcriptional network required for iNKT17 differentiation is not sufficient. This further supports Runx1’s role as a ‘key’ regulator for iNKT17 differentiation instead of simply a direct regulation of *rorc* expression during iNKT17 differentiation. However, it is not known whether Runx1 directly regulates the expression of BATF, c-Maf, Bcl11b and Lef1, or whether these are the only genes dysregulated during iNKT development in the absence of Runx1. To do this, future work will examine global changes in gene expression during iNKT cell development and differentiation in the absence of Runx1.

## Methods

### Mice

PLZF-cre^18^, Runx1^fl/fl^ ^56^, Rag1-GFP^43^, IL-7Rα Tg^57^, Lck-PLZF Tg^48^, and Lef1^fl/fl^ ^58^ mice has been previously described. All mice were on C57Bl/6 genetic background. Mice were housed in barrier facilities and all experiments were performed at the Mayo Clinic with the approval of the Institutional Animal Care and Use Committee. Mice of age 5–16 weeks were examined with either littermate or age-matched controls.

### Ethics approval

All animal experiments in this study were approved by the the Institutional Animal Care and Use Committee of Mayo Clinic and were performed in accrodance with relevant guidelines and regulations for animal experimentation at the Mayo Clinic.

### Flow Cytometry

All flow cytometry were performed on the Attune NxT (Life Technologies) or LSRII (BD Bioscience). Experiments were analysed using FlowJo (Tree Star). Each analysis had doublet cells excluded and live/dead gating using fixable viability dye (Tonbo) done before analysis, unless otherwise stated. Fluorophore conjugated or unconjugated antibodies were purchased from Tonbo, BD Bioscience, eBioscience, Biolegend, Cell Signaling Technology, AbCam or R&D Systems. The list of antibodies purchased is as follows: Tonbo: TCR-β (H57.597), CD4 (RM-5, GK1.5), NK1.1 (PK136), IL-4 (11311), IL-17A (TC11-18H10-1), IFN-γ (XMG1.2). eBioscience: Ki-67 (SolAl5), CD132 (TUGm2), IL-15R (DNT15Ra), Annexin V, CD24 (M1/69), NK1.1 (PK136), PLZF (Mags.21F7), Tbet (4B10), Bcl-2 (10C4), Runx1(RXDMC), Rat IgG2a,κ, (eBR2a), c-Maf (SYM0F1), mouse IgG2b,κ (eBMG2b). Biolegend: CD44 (IM7), CD8α (53-6.7), CD122 (TM-B1), CD24 (M1/69), IL-4 (11311), IL-17A (TC11-18H10-1), IFN-γ (XMG1.2), PLZF (Mags.21F7, 9E12), Tbet (4B10), Bcl-2 (10C4), CD4 (RM-5, GK1.5), CD127(A7R34). Cell Signaling Technologies: Mcl-1 (D2W9E), c-Myc (D84C12), pAKT(ser374) (193H12), BATF (D7C5), Lef1 (C12A5), Anti-Rabbit IgG (AF647), mTOR (7C10), pS6(D68F8). R&D: NK1.1 (KLRG-1C). AbCam: Bcl-xL (7B2.5), Bcl11b (25B6), Rat IgG2a,κ, (ab18446), BD Bioscience: ROR-γt (B2D), Runx3 (R3-5G4). CD1d tetramers loaded with PBS57 or empty were obtained from the NIH Tetramer Core Facility. Cells were stained with fixable viablity dye (FVD) in 1x PBS before staining for surface antigens in FACS buffer. For intracelluar stains, cells were stained with FVD and surface antigens before fixation and permeablization with Foxp3 fix:perm buffer kit (eBioscience or Tonbo) and then stained for intracellular antigens.

### Isolation of iNKT cells

Sorts were performed using a FACSAria (Becton Dickinson) in the Mayo Clinic Flow Cytometry Core Facility. Thymocytes were first stained with CD1d-PBS57 loaded tetramer (PE conjugated), then incubated with anti-PE coated magnetic beads (Miltenyi Biotec) to positively select iNKT cells. iNKT cells were then separated using (LS) MACS separation column. Isolated cells were then stained with FVD before surface stains with TCR-β, BV421-CD1d/PBS57 tetramer, CD24, CD44 and NK1.1 to distinguish iNKT cell developmental stages. Gated iNKT cells (PBS57-CD1d^+^ TCR-β^+^) were distinguished in stages: Stage 1 (CD24^−^ CD44^lo^ NK1.1^−^) Stage 2 (CD24^−^ CD44^hi^ NK1.1^−^) Stage 3 (CD24^−^ CD44^hi^ NK1.1^+^). iNKT cells were sorted directly into lysis buffer from RNeasy kit (Qiagen).

### Quantitative PCR

Using the RNeasy kit (Qiagen), mRNA from sorted iNKT cells were isolated. cDNA was generated and amplified using an Ovation PicoSL WTA V3 kit (NuGen) from mRNA isolated from sorted iNKT cells. *Runx1, nkap and Gapdh* Taqman assays used for q-PCR were purchased from Applied Biosystems. Assay used for *Runx1* spanned the deleted exon in the *Runx1* gene. Q-PCR was performed in ABI RT-PCR StepOne Plus System (Applied Biosystems) to calculate relative quantity (using 2^−ΔΔCT^ method, ref. 59) of *Runx1*. Relative *Runx1* expression in iNKT cells from PLZF-cre Runx1 cKO was calculated with WT Stage 3 (=1).

### Stimulation of iNKT cells

Stimulated iNKT cells were examined for cytokine production. Thymocytes were stimulated or unstimulated for total of 6 hrs in complete culture media (RPMI) with 200 nM PMA and 1uM Ionomycin. BFA/Monensin from eBioscience was added an hour after stimulation. Cell were harvested after additional 5 hours and washed before FACS staining. Cells were stained for FVD and surface antigens before fixed and permed to stain for cytokines and intracellular antigens.

### Apoptosis

Apoptotic iNKT cells were identified using FVD (Tonbo) and Annexin V (Biolegend) staining. Thymocytes were first stained as usual for FVD before surface stain. After surface stain, cells were washed and resuspended in 1x binding buffer then stained for Annexin V for 30 min on ice. Cell were washed and immediately analyzed by flow cytometry.

### Statistical analysis

Two-tailed Student’s *t* test was used for statistical analysis with *p* value < 0.05 considered significant.

## Electronic supplementary material

Supplemental Data
