# Supplementary material for: The differentiation of ROR-γt expressing iNKT17 cells is orchestrated by Runx1
Source: Sci Rep. 2017 Aug 1;7:7018. doi: 10.1038/s41598-017-07365-8 (PMC5539328; doi:10.1038/s41598-017-07365-8)
Supplement: Supplementary file 1 — Supplemental Data [file 41598_2017_7365_MOESM1_ESM.pdf]

Supplemental Information:

The differentiation of ROR-γt expressing iNKT17 cells is orchestrated by Runx1

Puspa Thapa[1], Bryce Manso[1], Ji Young Chung[1], Sinibaldo Romero Arocha[1], Hai-Hui Xue[2], Derek B. Sant' Angelo[3] and Virginia Smith Shapiro[1*]

[1]Department of Immunology, Mayo Clinic, College of Medicine, 200 1st Street SW, Rochester, MN 55905

[2]Department of Microbiology and Immunology, University of Iowa, 51 Newton Rd Iowa City, IA, 52242

[3]Department of Pediatrics, Rutgers Robert Wood Johnson Medical School and The Children's Health Institute of New Jersey, 89 French Street, New Brunswick, NJ 08901

[*]To whom correspondence should be addressed:
Virginia Smith Shapiro Ph.D.
200 1st Street SW
Rochester, MN 55905
Phone: 507-293-0615
Email: shapiro.virginia1@mayo.edu

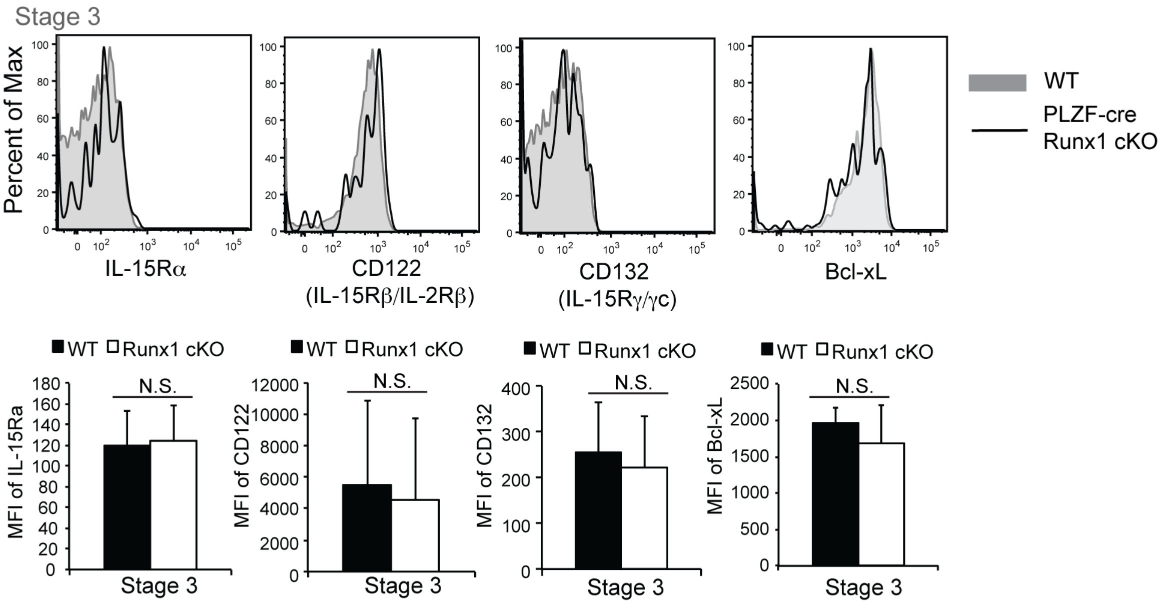

Supplemental Figure 1: Runx1-deficient Stage 3 iNKT cells have normal expression of IL-15R and Bcl-xL.

(**a**) Expression of IL-15 receptor subunits: IL-15Rα, IL-15Rβ (IL-2Rβ, CD122), IL-15Rγ (γc, CD132), and pro-survival gene Bcl-xL in Stage 3 iNKT cells of WT (grey filled) and PLZF-cre Hdac3 cKO (black line) mice. Data is representative of at least 3 mice/genotype from 3 independent experiments. Quantification of MFI for IL-15Rα, IL-15Rβ (IL-2Rβ, CD122), IL-15Rγ (γc, CD132), and Bcl-xL in Stage 3 iNKT cells of WT (black bar) and PLZF-cre Hdac3 cKO (white bar) mice. Data is calculated from at least 3 mice/genotype from 3 independent experiments. All statistical analysis was done using Student's *t-test.* Means ± S.E.M.

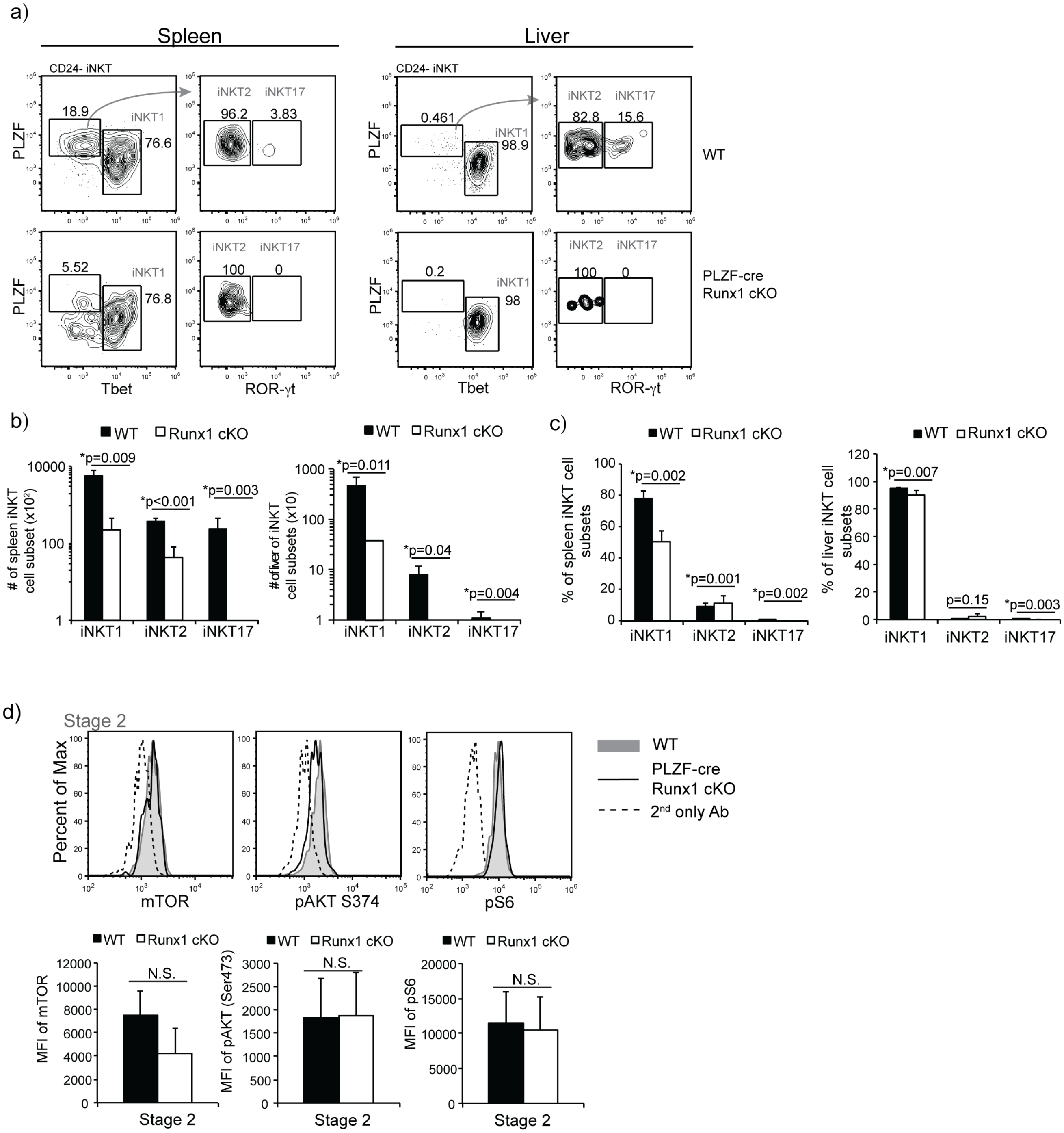

Supplemental Figure 2: Absence of peripheral iNKT17 cells in the spleen and liver of PLZF-cre Runx1 cKO mice.

(**a**) FACS analysis of splenic and liver iNKT1, iNKT2 and iNKT17 cells of WT (top) and PLZF-cre Runx1 cKO (bottom) mice. Gating for functional subsets were done as defined in Figure 4, using PLZF, Tbet and ROR-γt. Data is representative of at least 11 mice/genotype for spleen and 9 mice/genotype for liver. (**b**) Absolute number of iNKT cell subsets (iNKT1, iNKT2 and iNKT17) in spleen and liver of WT (black bars) and PLZF-cre Runx1 cKO (white bars) mice. Data is calculated from at least 11 mice/genotype for spleen and 9 mice/genotype for liver. (**c**) Frequency of iNKT cell subsets (iNKT1, iNKT2 and iNKT17) in spleen and liver of WT (black bars) and PLZF-cre Runx1 cKO (white bars) mice. Data is calculated from at least 11 mice/genotype for spleen and 9 mice/genotype for liver. (**d**) Expression of mTOR, pAKT (Ser374), and pS6 in Stage 2 iNKT cells of WT (grey filled), PLZF-cre Hdac3 cKO (black line) mice and Secondary antibody only control (dashed line). Data is representative of at least 4 mice/genotype from 4 independent experiments. Quantification of MFI for mTOR, pAKT (Ser374), and pS6 in Stage 2 iNKT cells of WT (black bar) and PLZF-cre Hdac3 cKO (white bar) mice. Data is calculated from at least 4 mice/genotype from 4 independent experiments. All statistical analysis was done using Student's *t-test.* Means ± S.E.M.

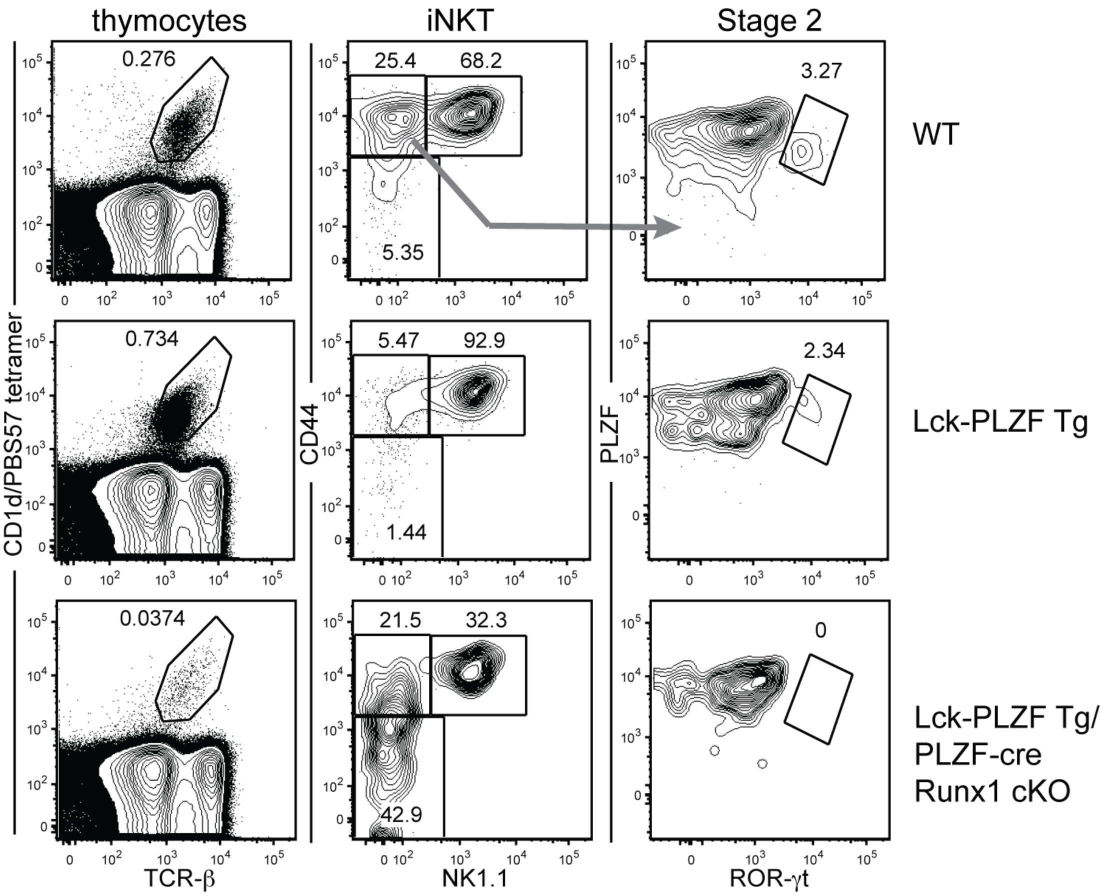

Supplemental Figure 3: Ectopic expression of PLZF in Runx1-deficient iNKT cells does not rescue block in iNKT17 differentiation. FACS analysis of iNKT cell development and ROR-γt expression in Stage 2 iNKT cells in WT, Lck-PLZF Tg and Lck-PLZF Tg/PLZF-cre Runx1 cKO mice. Analysis of iNKT cells was performed as described in Fig 1. Data is representative of 3 mice/genotype from 3 independent experiments.
